# Impact of Global Mean Normalization on Regional Glucose Metabolism in the Human Brain

**DOI:** 10.1155/2018/6120925

**Published:** 2018-06-12

**Authors:** Kristian N. Mortensen, Albert Gjedde, Garth J. Thompson, Peter Herman, Maxime J. Parent, Douglas L. Rothman, Ron Kupers, Maurice Ptito, Johan Stender, Steven Laureys, Valentin Riedl, Michael T. Alkire, Fahmeed Hyder

**Affiliations:** ^1^Department of Radiology & Biomedical Imaging and Magnetic Resonance Research Center, Yale University, New Haven, CT, USA; ^2^Department of Neuroscience, University of Copenhagen, Copenhagen, Denmark; ^3^Departments of Nuclear Medicine and Clinical Research, Odense University Hospital, University of Southern Denmark, Odense, Denmark; ^4^Department of Biomedical Engineering, Yale University, New Haven, CT, USA; ^5^Chaire de Recherche Harland Sanders, School of Optometry, University of Montreal, Montreal, Canada; ^6^Neuropsychiatry Laboratory, Psychiatric Centre, Rigshospitalet, Copenhagen, Denmark; ^7^GIGA-Consciousness, Coma Science Group, Université de Liège, Liège, Belgium; ^8^Departments of Neuroradiology, Nuclear Medicine and Neuroimaging Center, Technische Universität München, München, Germany; ^9^Department of Anesthesiology, University of California, Irvine, CA, USA

## Abstract

Because the human brain consumes a disproportionate fraction of the resting body's energy, positron emission tomography (PET) measurements of absolute glucose metabolism (CMR_glc_) can serve as disease biomarkers. Global mean normalization (GMN) of PET data reveals disease-based differences from healthy individuals as fractional changes across regions relative to a global mean. To assess the impact of GMN applied to metabolic data, we compared CMR_glc_ with and without GMN in healthy awake volunteers with eyes closed (i.e., control) against specific physiological/clinical states, including healthy/awake with eyes open, healthy/awake but congenitally blind, healthy/sedated with anesthetics, and patients with disorders of consciousness. Without GMN, global CMR_glc_ alterations compared to control were detected in all conditions except in congenitally blind where regional CMR_glc_ variations were detected in the visual cortex. However, GMN introduced regional and bidirectional CMR_glc_ changes at smaller fractions of the quantitative delocalized changes. While global information was lost with GMN, the quantitative approach (i.e., a validated method for quantitative baseline metabolic activity without GMN) not only preserved global CMR_glc_ alterations induced by opening eyes, sedation, and varying consciousness but also detected regional CMR_glc_ variations in the congenitally blind. These results caution the use of GMN upon PET-measured CMR_glc_ data in health and disease.

## 1. Introduction

Noninvasive neuroimaging with positron emission tomography (PET) and functional magnetic resonance imaging (fMRI) provide the foundations of human brain mapping, as practiced in the past four decades for PET and three decades for fMRI [1–4]. Early PET studies concentrated on quantitative imaging of resting-state blood flow and metabolism [2, 5], whereas later PET and then fMRI studies used tools with some form of global mean normalization (GMN), most notably statistical parametric mapping (SPM), or the Scaled Subprofile Model of principal component analysis (SSM-PCA), to obtain regional differences among control and metabolically/functionally perturbed states. When these methods are applied to PET metabolic radiotracers, such as [^18^F]fluorodeoxyglucose (FDG), the application of these analysis tools often proceeded with the assumption that global brain metabolic activity, defined as the mean metabolic rate of gray matter or the entire brain, is a valid basis for normalization of regional values, in part because it is held to facilitate group comparisons in the presence of physiological and/or experimental intraindividual and interindividual differences [6, 7]. GMN yields parametric images of fractional or percentage differences from a variably defined global mean. While it is not a formal requirement for either SPM or SSM-PCA, GMN has become an almost routine and a necessary preparatory step in analyses of neuroimaging data. For example, in the SSM-PCA method, the global effects are removed by log transformation and centering, but this procedure has a similar effect to GMN in that it removes any scalar multiplicative parameters at the individual level. Many PET studies exemplify the use of GMN to reveal differences of cerebral metabolic rate of glucose (CMR_glc_) across states of health and different diseases. However, to validate the metabolic differences, it is necessary to adequately account for the substantial variability of resting metabolic rates of human brain among individuals and brain states [8].

The covariance pattern extraction can be independent of the normalization process on PET data. For example, SSM-PCA analysis does not proceed as a form of GMN of the data. Steps like log transformation and centering can, on certain conditions, be used to remove scalar factors from the underlying disease patterns, which may be caused by variable spatial covariance. Thus, it is important to differentiate univariate assessment of CMR_glc_ from voxel weights of disease pattern derived as principal components of multivariate spatial covariance. Effects of absolute quantification on SSM derivation of disease-specific network profiles were reported by Strother et al. [9] and more recently by Borghammer et al. [10–14], who concluded that GMN can yield spurious interpretations of perturbed measures of brain activity. Commonly used PCA analysis methods focus on regional differences at the group level, beyond differences in global brain function. However, we contest that analysis methods which assume that global differences do not exist may cause global effects to contaminate “local” results, and thus, global effects should be separately evaluated to avoid this concern.

Several decades after the advent of PET, fMRI became the common method of choice to detect functional differences among brain regions and/or, differences between control and patient groups, and for mapping of functional connectivity in resting brain [15]. The fMRI approach reveals resting-state correlations of the somewhat poorly defined regional blood oxygenation level-dependent (BOLD) signal among brain regions. Thus “functional connectivity networks” are derived by searching for significant correlations from the spontaneous fluctuations of the BOLD signal. However, the BOLD signal itself is a nontrivial function of oxygen extracted from the circulation and, therefore, reflects changes in rates of both cerebral blood flow (CBF) and oxidative metabolism (CMR_O2_) [16]. Most analysis methods of resting-state fMRI remove, among other variables, the global BOLD signal to reveal the networks [17]. Results are then inferred within these so-called resting-state networks from the remaining fluctuations, where the amplitude of the spontaneous BOLD signal is significantly reduced upon regression [17, 18]. In contrast, FDG-PET reveals resting-state network activity by calculation of differences among the regional CMR_glc_ of a group of subjects or across different metabolic states, with subsequent application of network analysis to the regions that differ among groups and/or conditions [19]. Most resting-state fMRI and PET studies thus use some form of GMN prior to comparison of the data for network determination. However, our focus here is only the effects of GMN upon PET imaging.

The validity of the GMN procedure for creating metabolic maps originally remained uncontested on the assumption that most glucose and oxygen consumed in the resting-state served “nonfunctional” mechanisms which are uncorrelated with cognitive activity [20]. However, results from both early and more recent studies challenge this assumption [18, 21]. The resting brain is the most energy-demanding organ in the human body [22], the energy turnover due to Na^+^,K^+^-ATPase function that sustains membrane repolarization and ion gradient restoration for continuous neuronal activity [23–25]. It is well accepted that energy demands of neuronal activity in the resting awake human brain by far exceed the magnitude of the additional energy turnover associated with evoked or spontaneous changes of functional activity [26]. Yet, the fraction of the total metabolic rate altered by spontaneous or evoked events remains uncertain, and the extent to which GMN obscures differences of the energy demand across functional states thus still remains uncertain. In this context, CBF and CMR_O2_ values measured in healthy aging and in Parkinson's disease show that conventional GMN obscures evidence of metabolic changes in the brain [27, 28]. Borghammer et al. showed that GMN of quantitative PET-measured CBF measurements can yield false positive findings of perfusion changes [10], but the methods are nonetheless being generalized to metabolic PET scans [29]. Borghammer et al. also demonstrated that foci of elevated CBF attributed to small brain regions actually can arise as a consequence of normalization applied only to gray matter [11]. In an examination of simulated reduction of cortical metabolism, Borghammer et al. further noted that GMN generally only recovered a few percent of the original signal and conversely led to artifactual findings of relative increases [12]. Thus, there are two issues that potentially affect the use of PET images as biomarkers of disease; the raising of regional differences to significance and the removal of global differences among individuals and groups that results from GMN.

Prompted by this evidence, we sought to test the hypothesis that GMN may not only artificially raise minor regional variations to significance but also may significantly obscure global metabolic effects when PET images of the resting brain in specific disorders are compared. We used FDG-PET to measure CMR_glc_ at different sites, where the control states (of resting healthy awake volunteers with eyes closed) were compared to subjects in states established by conditions ranging from normal sensory input to sedation by anesthesia to different clinical states. While some experiments involved blood sampling of the FDG tracer's supply to the brain, necessary to obtain absolute values of CMR_glc_ (aCMR_glc_), others did not. To compare FDG-PET images from different sites, we developed a new method allowing quantitative measures of CMR_glc_ (qCMR_glc_) by a calibration procedure that is based on comparison of qCMR_glc_ data with aCMR_glc_ data for a control state (i.e., healthy awake with eyes closed). We then validated the method, which is aimed for quantitative baseline metabolic activity without GMN, by first comparing qCMR_glc_ values found in control experiments from different sites and then comparing qCMR_glc_ to aCMR_glc_ for experiments with blood sampling. We tested conditions that included awake and eyes open states (presence of sensory input), pharmacological intervention (anesthesia), disorders of consciousness, and congenital blindness (clinical states), in comparison to resting healthy awake subjects with eyes closed (control). Comparison of *t*-maps of CMR_glc_ without GMN reveal heuristically important and pathognomonic evidence of perturbations of brain metabolism across states or among groups. However, GMN induced artificial relative increases in states that are generally accepted as only inducing metabolic decreases.

## 2. Materials and Methods

### 2.1. Subjects

Participants underwent tomography at four sites, and imaging at each site included a control group. FDG-PET measures were collected in a total of nine different resting states (Table 1) and compiled as anonymized data, most of which previously had been published prior to the present analysis. A group of healthy awake subjects imaged with eyes closed (HAEC) was recorded at each site. Each site's HAEC served as control for the other groups recorded at that site. There were 8 other groups: healthy awake subjects with eyes open (HAEO) [30]; healthy subjects sedated with 1% desflurane (Des1%), 0.25% sevoflurane (Sev0.25%) [31], or 0.5% sevoflurane (Sev0.5%); awake congenitally blind (CB) subjects [32]; and patients with disorders of consciousness, including unresponsive wakefulness syndrome (UWS), minimally conscious state (MCS), and emergence from MCS (EMCS) [33]. The diagnostic criteria for the selected disorders of consciousness have been described earlier [34]. All healthy participants were right-handed.

Among the five groups of healthy volunteers, two without sedation (HAEC and HAEO) underwent tomography in Munich, Germany, and those with sedation (Sev0.25%, Sev0.5%, and Des1%) in Irvine, CA, USA. Among the four groups with some form of disability, the CB underwent tomography in Copenhagen, Denmark. All three groups with disorders of consciousness had tomography in Liège, Belgium. All tomograms were acquired upon obtaining written informed consent from participants or from caregivers (in the case of disorders of consciousness), in accordance with the Helsinki Protocol, and all studies were approved by the appropriate ethical review board per institution; the Ethics Committee of the University Hospital of Liège (Belgium), the Research Ethics Committee of the University of Copenhagen and Frederiksberg (Denmark); the Institutional Review Board at the University of California, Irvine (USA); and the ethics review board of the Klinikum Rechts der Isar, Technische Universität München (Germany).

### 2.2. Tomography

All subjects underwent FDG-PET and MRI scanning. Details of FDG-PET and MRI acquisition are described in the original studies [30–34]. Briefly, tomographies in USA and Denmark were performed on Siemens ECAT high-resolution research tomographs (HRRT), in Germany on a Siemens Biograph mMR PET/MRI, and in Belgium on a Philips GEMINI TF PET/CT. Blood sampling in USA subjects allowed calculation of absolute values of CMR_glc_ (aCMR_glc_) [31].

#### 2.2.1. Tomography (Site Number 1)

The two healthy groups without any sedation, consisting of 11 HAEO subjects (aged 52 ± 10 years, 7 males) and 11 different HAEC subject (aged 57 ± 10 years, 8 males; i.e., HAEC_GER_), all used an MRI/PET tomograph (Siemens Biograph mMR) at the Neuroimaging Center of Technical University of Munich, Germany (Table 1). Subjects held their eyes closed or open depending on their assigned group; details of the scans have been published elsewhere [30]. Structural MRI data were acquired (magnetization-prepared 180-degree radiofrequency pulses and rapid gradient-echo (MP-RAGE), repetition time (TR) 2.3 s, echo time (TE) 2.98 ms, 160 slices with 0.5 mm gap, 256 × 256 mm field of view (FOV), 256 × 256 matrix size, and 5 minutes and 3 seconds). About 30 minutes after the bolus FDG injection, a 10-minute emission recording was acquired (saturated list mode, 128 slices with 0.5 mm gap, 192 × 192 mm matrix, and 3.7 × 2.3 × 2.7 mm voxel).

#### 2.2.2. Tomography (Site Number 2)

The three sedated groups (age range 18–22 years), consisting of 8 Sev0.25% subjects, 8 Sev0.5% subjects (same cases as Sev0.25%), and 7 Des1% subjects, were all scanned using the Siemens ECAT high-resolution research tomograph (HRRT) at the Department of Anesthesiology of the University of California, Irvine, California, USA, and also underwent MRI (Table 1). Details of the tomographies of the Sev0.25% group, same as the other groups, have been published elsewhere [31]. Two intravenous catheters were inserted, one for arterialized venous blood sampling and the other for FDG infusion (203.5 MBq) enabling measurement of absolute CMR_glc_. A brief attenuation scan was obtained using a Cs-137 source, and a ten-minute emission recording was obtained (207 slices at 1.2 mm gap) beginning 32 min after FDG application; participants were still for the tracer uptake interval, except when asked to perform a hand gesture as a test of alertness/sedation. The tomograph had an effective resolution of 3.3 mm full width at half maximum (FWHM). Participants had tomographies on different occasions for the selected doses of anesthetic gases, delivered with standard calibrated vaporizers in 100% oxygen via a standard semicircle breathing circuit using a Dräger AV anesthesia machine. A Datex Ohmeda Capnomac Ultima (Helsinki, Finland) was used to monitor expired CO_2_ and anesthetic gas levels. This HAEC group consisted of the participants who received 0% sevoflurane (HAEC_sev_; *n* = 8; Table 1) and 0% desflurane (HAEC_des_; *n* = 7; Table 1).

#### 2.2.3. Tomography (Site Number 3)

PET data were acquired in a group of 7 CB participants (three males aged 41 ± 8 years) and 7 HEAC (aged 25 ± 5 years, four males; HAEC_DEN_) using a Siemens ECAT HRRT at Rigshospitalet in Copenhagen, Denmark (Table 1). Participants' MRIs were acquired using a 3 T Siemens Trio MRI scanner at the Danish Research Centre for Magnetic Resonance, Hvidovre Hospital, Hvidovre, Denmark. Details of the scans have been published elsewhere [32]. One among the seven CB participants had limited vision at birth that progressed to complete blindness at the age of seven; all others were completely blind from birth. Structural MRI data were acquired (MP-RAGE, TR 1.5 s, TE 3.93 ms, inversion recovery time (TI) 0.8 s, 256 slices with no gap, 192 × 256 mm FOV, and 6 minutes 36 seconds). PET data were acquired forty minutes after bolus injection of approximately 210 MBq FDG (single frame, OSEM3D mode, 207 slices with no gap, 1.2 × 1.2 × 1.2 mm voxels, and 40 minutes). During the tracer uptake period, control participants were blindfolded and all participants rested in a dimly lit room without falling asleep.

#### 2.2.4. Tomography (Site Number 4)

The groups with disorders of consciousness consisted of (i) 49 UWS patients (aged 46 ± 16, 31 males; mean time since injury 1.7 ± 3.2 years), (ii) 65 MCS patients (aged 40 ± 16, 41 males; mean time since injury 3.3 ± 4.3 years), and (iii) 17 EMCS patients (aged 35 ± 15, 15 males; mean time since injury 3.0 ± 3.7 years). The control group (HAEC_BEL_) consisted of 28 participants (aged 44 ± 16, 16 males). All participants were scanned using the Philips GEMINI TF PET/CT device at the University Hospital of Liege, Liege, Belgium (Table 1), according to procedures described in detail elsewhere [33–35]. About 30 min after intravenous FDG injection, a single 12-minute emission frame was recorded (90 slices with no gap, 256 × 256 matrix, and 2 × 2 × 2 mm voxels). The control subjects were kept awake in a dimly lit room during the FDG uptake, and all patients were kept awake during FDG uptake.

### 2.3. Registration

All PET images were registered to the Montreal Neurological Institute (MNI) space (3 × 3 × 3 mm) using a combination of linear and nonlinear registration tools on publicly available platforms (i.e., advanced normalization tools (ANTs) from http://stnava.github.io/ANTs, or BioImage Suite from http://bioimagesuite.yale.edu). PET images from Germany, USA, and Denmark were first registered to their corresponding MRI image using a rigid body transformation and then carried to the MNI template by computed affine and nonlinear transformations, with interpolation to a 3 × 3 × 3 mm^3^ voxel size. Belgian PET images were directly registered to a common PET template created from the HAEC_DEN_ group, using a combination of linear and nonlinear registrations applying very restrictive and highly regularized registration parameters.

### 2.4. Calibrating Quantified Measures of CMR_glc_ (qCMR_glc_)

As shown in Table 1, only the USA site had blood sampling data to enable FDG-PET counts to be converted into “absolute CMR_glc_” units of *μ*mol/g/min (aCMR_glc_). To compare metabolic measurements recorded from different sites (where blood sampling data were unavailable), we developed a new method for calibrating quantified measures of CMR_glc_ (qCMR_glc_) that targets quantitative baseline metabolic activity without GMN. This method is based on the comparison of qCMR_glc_ data with aCMR_glc_ data also for the HAEC condition from Hyder et al. study (aCMR_glc_-HYD), with a mean male age of 26.1 ± 3.8 years [36]. For consistency of the data from the USA site with data from other sites, we also calculated qCMR_glc_ for these five USA datasets, which in turn provided the validation for our procedure (see below).

Our goal was to preserve between-state global differences in metabolism, which are believed to be removed by GMN. Previous work has demonstrated that for identical conditions (i.e., HAEC), region-to-region aCMR_glc_ variation is proportional to region-to-region PET radiation counts [37]. We opted to apply per-site fitting procedure by using the same linear model for all individuals at a given site. Assuming HAEC groups are comparable across sites [19], then this procedure would have the potential to compare metabolic differences between states recorded at different sites.

The “quantified CMR_glc_” metric, referred to as qCMR_glc_ to focus on quantitative baseline metabolic activity without GMN, was obtained in two steps. First, a linear intensity transformation of the original tissue radioactivity values was computed on a per-site basis, such that the distribution of voxels in the mean across the gray and white matter of the cerebrum (excluding the cerebellum) from each site was matched in intensity to the distribution of voxels from the published aCMR_glc_-HYD database [36]. The similarity between the distributions was calculated as the Jensen-Shannon Divergence [38] (JSD), where the per-site linear intensity transformation was calculated as the minimization of the following expression:

(1) $$\begin{array}{c} JSD\left[dist\left(<{aCMR}_{glc}-HYD>\right)–dist\left(a_{site}\cdot <{FDG}_{HAEC}>+b_{site}\right)\right], \end{array}$$

where dist (<aCMR_glc_ − HYD>) and dist (<FDG_HAEC_>), respectively, refer to the distribution of voxels in the mean across the gray and white matter of the cerebrum (excluding the cerebellum) of the published aCMR_glc_-HYD database [36] and the original tissue-radioactivity values for each HAEC group (FDG_HAEC_) from any site (Table 1), and *a*_site_ and *b*_site_ are, respectively, the resultant slope and intercept from the fit, unique for the specific site. Prior to minimization of eq. (1), <FDG_HAEC_> was spatially smoothed to match the point-spread function of <aCMR_glc_-HYD> as computed by the 3dFWHMx program from the AFNI software package. Then, the qCMR_glc_ maps for each subject were computed by applying the *a*_site_ and *b*_site_ from eq. (1) as follows:

(2) $$\begin{array}{c} {qCMR}_{glc}=a_{site}\cdot FDG+b_{site}, \end{array}$$

where FDG refers to tissue-radioactivity concentrations from any individual voxel for any single subject in any group and only for the specific site for which *a*_site_ and *b*_site_ were calculated. The calculated qCMR_glc_ was used throughout this study as fitted between each site's HAEC group and all other groups from that site. The qCMR_glc_ calculation was carried out using the distributions of only intracranial voxels.

Two tests were run to validate qCMR_glc_. First, if comparable qCMR_glc_ values exist in HAEC groups from different sites, this would indicate that between-site comparisons are possible. To test this, the mean qCMR_glc_ within 41 gray matter regions (Table S1) drawn in the MNI reference space was calculated for the five control groups (HAEC_DEN_, HAEC_GER_, HAEC_sev_, HAEC_des_, and HAEC_BEL_) and aCMR_glc_-HYD from Hyder et al. [36] that also represented the HAEC condition. Pearson correlation and Euclidean distance were calculated between the group means of aCMR_glc_-HYD and qCMR_glc_ in their respective 41 gray matter regions repeated for each pair of groups. Then, *p* values for statistical significance were calculated with permutation testing across the 41 gray matter regions with 1000 repetitions and rerunning the correlation and distance calculations then taking the percentile of the actual correlation/distance based on the randomly permuted correlations/distances as a null distribution (one-sided test, Pearson correlation higher than the null hypothesis and Euclidean distance lower than the null hypothesis).

Second, as a further test of the ability to compare qCMR_glc_ between groups, we used aCMR_glc_ data that was available from the USA site. The Des1% and HAEC_des_ groups had the same subjects, as did the Sev0.25%, Sev0.5%, and HAEC_sev_ groups. The same data were also used to calculate qCMR_glc_ (see above). Means of aCMR_glc_ and qCMR_glc_ were calculated within each gray matter region across all subjects. Treating the respective HAEC group as the *x*-axis and the respective anesthetized group as the *y*-axis, a linear fit was calculated. The slopes from the linear fits from aCMR_glc_ were compared to those from qCMR_glc_ to establish the validity of our calibration method.

### 2.5. Image Analysis

Mean qCMR_glc_ maps were computed as the voxel-by-voxel average across each group of subjects and for the combined group of control subjects from all tomography sites. Statistical *t*-maps were computed using an unpaired voxel-wise two-sample two-tailed Student's *t*-test, assuming equal variance for qCMR_glc_ images following smoothing with an 8 mm Gaussian kernel. Statistical *t*-maps were also generated with the same parameters following GMN images, with individual scaling to the whole-brain mean of qCMR_glc_. Statistics were computed both for qCMR_glc_ and GMN images using the gray matter regions (Table S1) for difference of each state from the HAEC condition.

## 3. Results

### 3.1. Validating Quantified Measures of CMR_glc_ (qCMR_glc_)

**Figure 1:**
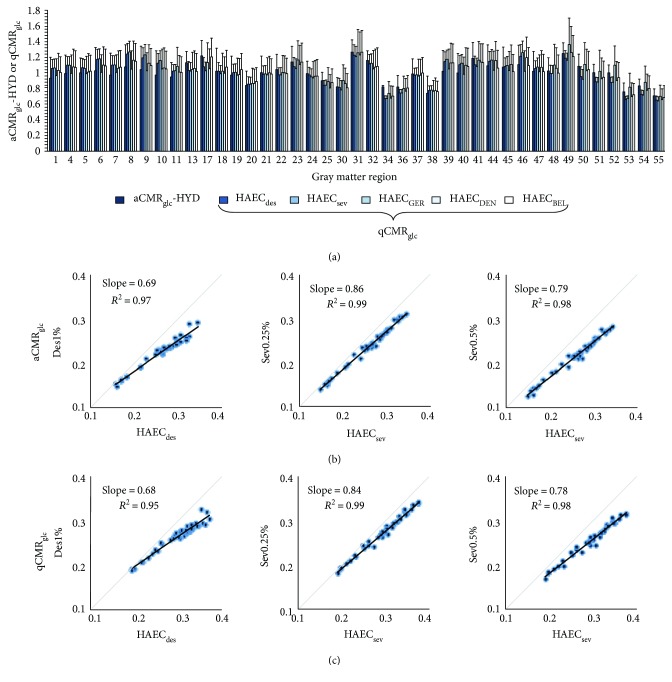
Validation of quantified CMR_glc_ (qCMR_glc_). (a) Comparison of absolute CMR_glc_ from Hyder et al. [36] (aCMR_glc_-HYD) with qCMR_glc_ from five sites for the HAEC condition (Table 1). Bars represent the mean across all subjects in each group for gray matter regions (Table S1), where error bars are one standard deviation. All qCMR_glc_ and aCMR_glc_-HYD values were very similar both relatively between regions and in terms of mean value, suggesting across-site comparisons are possible with our procedure for quantified CMR_glc_. The Pearson correlation and the Euclidean distance (Table 2) suggest high similarity and low difference of CMR_glc_ for the HAEC group across all sites. (b) Scatter plots, from left to right, for aCMR_glc_ between Des1%, Sev0.25%, or Sev0.5% groups and equivalent HAEC groups (Table 1). Each point is one gray matter region (Table S1). Slope and *R* ^2^ from a linear fit are shown, and units are *μ*mol/g/min, where a slope of less than 1 corresponds to a lower CMR_glc_ in the anesthetized group compared to the control. (c) Same as in (b), except using qCMR_glc_ from each group. The slopes are almost identical in (b) and (c), indicating that the calculation of qCMR_glc_ does not alter the relationship between groups for aCMR_glc_.

We compared the mean quantified estimates of CMR_glc_ (qCMR_glc_) across 41 gray matter regions for the five HAEC groups listed in Table 1 to absolute CMR_glc_ (aCMR_glc_) of the HAEC group from Hyder et al. [36] (aCMR_glc_-HYD), as shown in Figure 1(a). The Pearson correlation and Euclidean distance between each pair of groups are listed in Table 2. All correlations were highly significant, and Euclidean distances were less than half of the mean in even one dimension, despite there being 41 dimensions. Although HAEC_sev_ and HAEC_des_ correlated the highest because the subjects in these groups overlapped, different groups of subjects (e.g., HAEC_des_ and HAEC_DEN_ or HAEC_sev_ and HAEC_DEN_) had similarly high correlation. We attribute the high correlation among control subjects to the tight age group. The *p* values resulting from comparing actual correlations and distances to an artificially generated null distribution were zero (for Pearson correlation, the value is higher than for 1000 random permutations; for Euclidean distance, the value is lower than for 1000 random permutations). The values indicate that all HAEC groups, whether associated with aCMR_glc_ or qCMR_glc_, were highly similar in terms of both spatial extent (correlation) and actual value (distance), confirming that it is valid to compare the HAEC groups from different sites.

Figure 1(b) shows the linear fit between the states of anesthesia and respective control states using the aCMR_glc_ estimates from site number 2, while Figure 1(c) shows the same fit for qCMR_glc_ estimates where a slope of less than 1 in both Figures 1(b) and 1(c) corresponds to a lower qCMR_glc_ in the anesthetized group compared to the control group. All linear fits had *R* ^2^ ≥ 0.95. The slopes of the linear fits were nearly identical for aCMR_glc_ and qCMR_glc_ (Des1%: 0.69 versus 0.68; Sev0.25%: 0.86 versus 0.84; and Sev0.5%: 0.79 versus 0.78). We also noted small but consistent shifts of intercepts between the healthy awake and sedated, which were reproducible for aCMR_glc_ and qCMR_glc_ (Des1%: 0.045 versus 0.062; Sev0.25%: 0.013 versus 0.024; and Sev0.5%: 0.011 versus 0.0.024). The largely consistent slope estimates for aCMR_glc_ and qCMR_glc_ in Figures 1(b) and 1(c) demonstrate that group-to-group differences present in aCMR_glc_ estimates were preserved after calculating qCMR_glc_.

### 3.2. qCMR_glc_ across Different States

**Figure 2:**
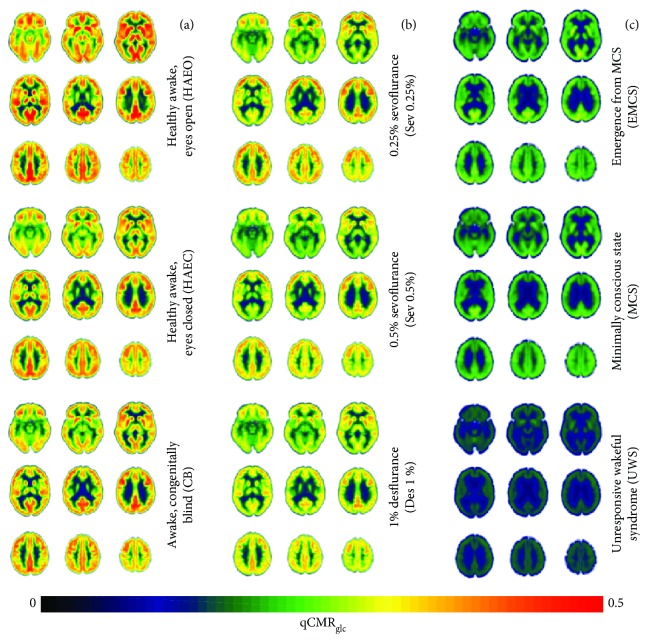
Quantified CMR_glc_ (qCMR_glc_) maps of the human brain across nine different states (Table 1), which are (a) healthy awake sighted people with eyes closed (HAEC) or with eyes open (HAEO), as well as healthy awake people who are congenitally blind (CB), (b) healthy people under sedation with gaseous anesthetics (i.e., 0.25% sevoflurane (Sev0.25%), 0.5% sevoflurane (Sev0.5%), and 1% desflurane (Des1%)), and (c) patients with disorders of consciousness (i.e., unresponsive wakefulness syndrome (UWS), minimally conscious state (MCS), and emergence from minimally conscious state (EMCS)). Global increases in qCMR_glc_ were observed proceeding from bottom to top in each column, which is in general agreement with prior PET studies [19, 45–50, 53]. The units are in *μ*mol/g/min. With HAEC as the control condition, all other groups (except the CB group) showed significant global differences in gray matter (Figure S1) and white matter (Figure S2). These and all other images are in the coordinates of the MNI template: left column (from top to bottom) with *z* values of −15 mm, −12 mm, and −39 mm; middle column (from top to bottom) with *z* values of −6 mm, −21 mm, and −48 mm; and right column (from top to bottom) with *z* values of −3 mm, −30 mm, and −57 mm.

Compared to the eyes closed condition of the HAEC control group members (0.31 ± 0.06 *μ*mol/g/min), the eyes open HAEO group members had higher global estimates of qCMR_glc_ (0.34 ± 0.06 *μ*mol/g/min) and the CB members had similar global gray matter estimates of qCMR_glc_ (0.31 ± 0.05 *μ*mol/g/min), as shown in Figure 2(a). The HAEO group members had 8–12% higher global qCMR_glc_ estimates (0.34 ± 0.06 *μ*mol/g/min) compared to the members of the HAEC control group in both gray and white matter regions (Figures S1A and S2A, resp.). The qCMR_glc_ differences between HAEC and HAEO match reports of simple radiation counts [37]. In contrast, members of the CB group revealed only insignificant differences of global qCMR_glc_ estimates across gray and white matter regions, compared with members of the HAEC groups (Figures S1B and S2B, resp.).  Table 3 shows the relationship of qCMR_glc_ when comparing different states to the control condition as assessed by linear regression analysis with intercept at zero (intercept = 0) and a floating intercept (intercept ≠ 0). Compared to HAEC, decreasing slopes were observed from HAEO to CB to Sev0.25% to Sev0.5% to Des1% to EMCS to MCS to UWS in both gray and white matter, and this pattern did not change with the regression method. There were minimal differences in the slopes (less than 16%) between the two regression methods except for UWS, which also had the largest intercept (0.07 in gray matter and 0.05 in white matter). The intercepts in all other cases were much smaller in comparison, suggesting that intercept at zero is a sufficient approximation for most of the states examined (Figures S1 and S2).

Compared to HAEC controls, the groups of individuals under sedation (Sev0.25%, Sev0.5%, and Des1%) had lower global qCMR_glc_ estimates (0.29 ± 0.06, 0.27 ± 0.05, and 0.27 ± 0.05 *μ*mol/g/min in gray matter, respectively; Figure 2(b)). Compared to the HAEC control group members, members of the three sedation groups had 8–15% lower qCMR_glc_ estimates in gray matter (Figure S1C) and 8–12% lower estimates in white matter (Figure S2C).

Compared to the HAEC group of control subjects, patients with disorders of consciousness (UWS, MCS, and EMCS) all had significantly lower qCMR_glc_ estimates (0.20 ± 0.04, 0.19 ± 0.04, and 0.14 ± 0.02 *μ*mol/g/min in gray matter, resp.; Figure 2(c)). Compared to the HAEC control group, the clinical states had 36–54% lower estimates of qCMR_glc_ in gray matter (Figure S1D) and 29–43% lower estimates in white matter (Figure S2D).

### 3.3. Statistical *t*-Maps for qCMR_glc_ and GMN Data across States

**Figure 3:**
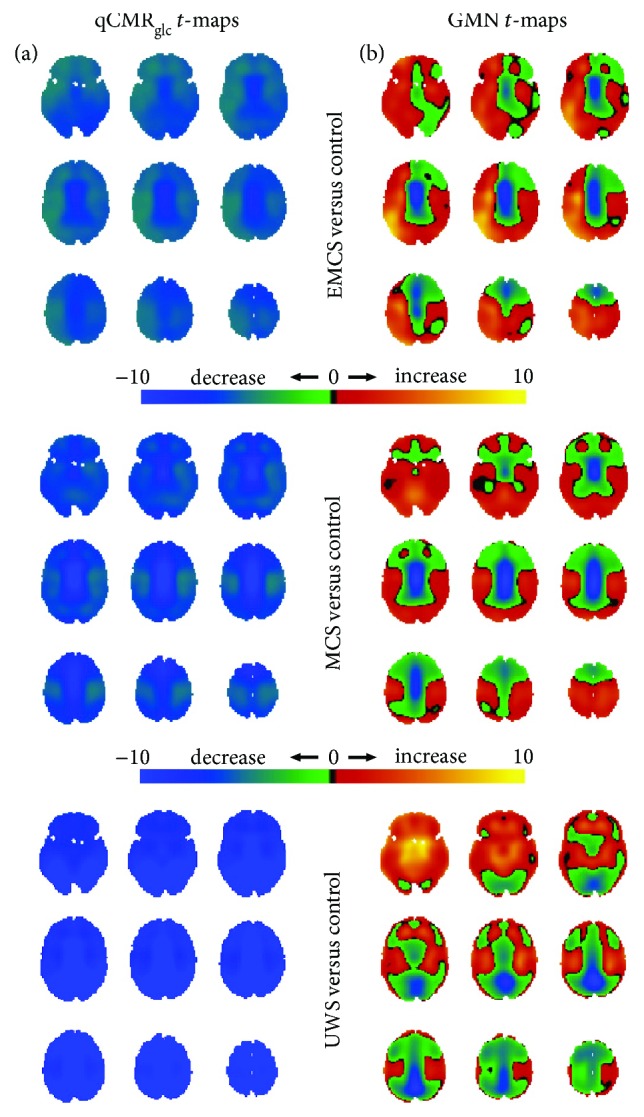
Spatial distributions of metabolic variations in patients with disorders of consciousness (i.e., UWS, MCS, and EMCS in Figure 2(c)) versus the control group (i.e., HAEC in Figure 2(a)), shown with respect to unthresholded Student's *t*-maps using (a) qCMR_glc_ images and (b) GMN images. (a) For the UWS, MCS, and EMCS groups, the unthresholded *t*-maps with qCMR_glc_ indicated globally unidirectional metabolic decreases in patients with disorders of consciousness. (b) But the unthresholded *t*-maps with GMN demonstrated the presence of regionally bidirectional metabolic changes in disorders of consciousness. Based on validation of qCMR_glc_ to aCMR_glc_-HYD (Figures 1 and 2; Table 2), without GMN the global decreases corresponded to about 0.15 *μ*mol/g/min (UWS < MCS ≈ EMCS) and with GMN the global changes were diminished to put overemphasis on the regional differences. See Figure S3 for thresholded maps (Table 4).

**Figure 4 fig4:**
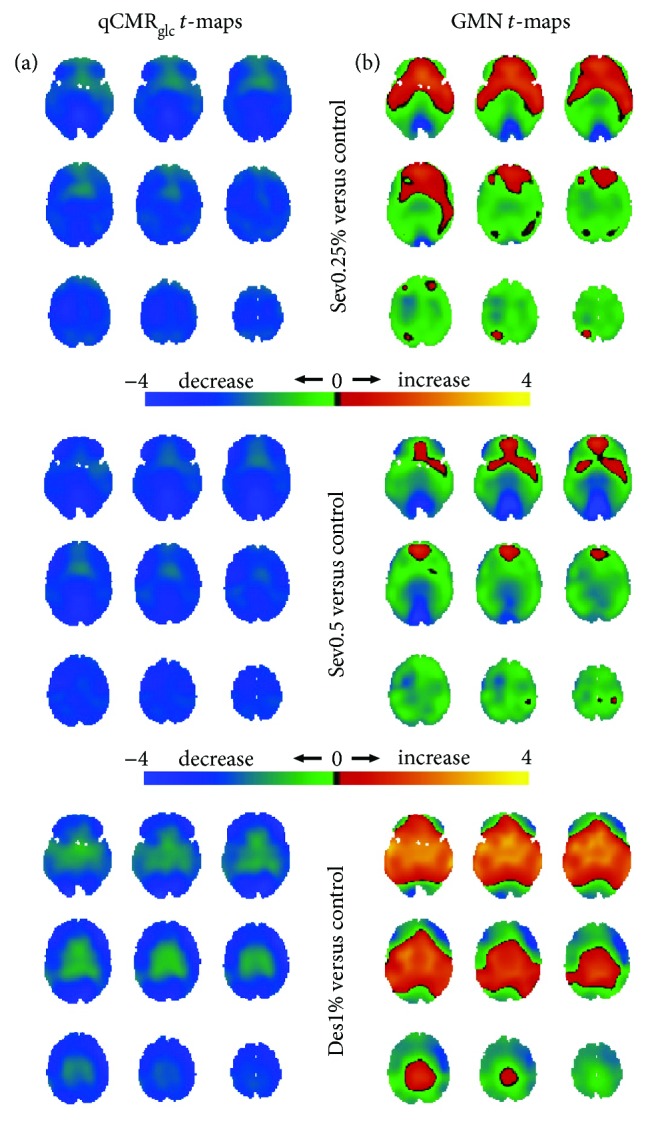
Spatial distributions of metabolic variations with sedation (i.e., Des1%, Sev0.25%, and Sev0.5% in Figure 2(b)) versus the control group (i.e., HAEC in Figure 2(a)), shown with respect to unthresholded Student's *t*-maps using (a) qCMR_glc_ images and (b) GMN images. (a) For Des1%, Sev0.25%, and Sev0.5% groups, the unthresholded *t*-maps with qCMR_glc_ indicated globally unidirectional metabolic decreases with sedation. (b) However, the unthresholded *t*-maps with GMN depicted regions with metabolic increases and decreases upon sedation. Based on validation of qCMR_glc_ to aCMR_glc_-HYD (Figures 1 and 2; Table 2), without GMN the global decreases corresponded to about 0.05 *μ*mol/g/min (Des1% ≈ Sev0.5% < Sev1%) and with GMN the deemphasis on global changes put the focus on the regional differences. See Figure S4 for thresholded maps (Table 4).

**Figure 5 fig5:**
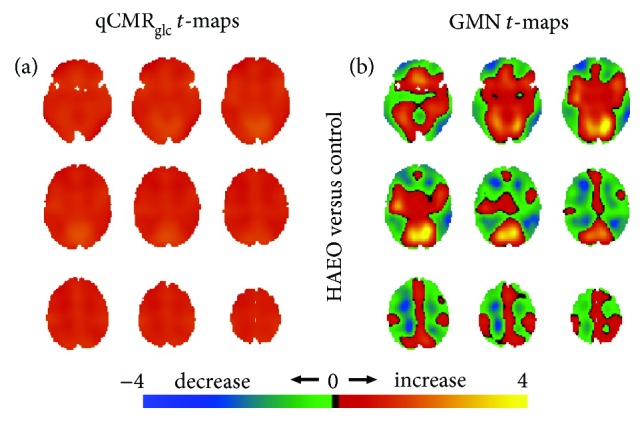
Spatial distributions of metabolic variations with healthy participants with eyes open (i.e., HAEO in Figure 2(a)) versus the eyes closed control group (i.e., HAEC in Figure 2(a)), shown with respect to unthresholded Student's *t*-maps using (a) qCMR_glc_ images and (b) GMN images. (a) For the HAEO group, the unthresholded *t*-maps with qCMR_glc_ indicated the presence of globally unidirectional metabolic increases with eyes open. (b) Conversely, the unthresholded *t*-maps with GMN revealed regions of increased and decreased metabolism with eyes open. Based on validation of qCMR_glc_ to aCMR_glc_-HYD (Figures 1 and 2; Table 2), without GMN the global increases corresponded to about 0.05 *μ*mol/g/min while with GMN, the global changes were minute. See Figure S5 for thresholded maps (Table 4).

**Figure 6:**
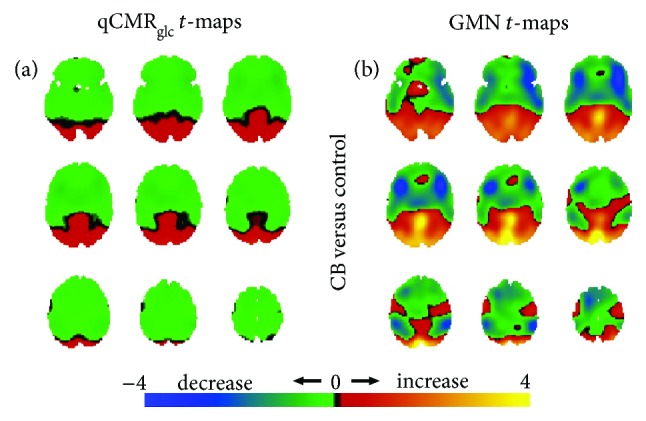
Spatial distributions of metabolic variations in the congenitally blind (i.e., CB in Figure 2(a)) versus the eyes closed control group (i.e., HAEC in Figure 2(a)), shown with respect to unthresholded Student's *t*-maps using (a) qCMR_glc_ images and (b) GMN images. (a) For the CB group, the unthresholded *t*-maps with qCMR_glc_ images indicated both regions with increased and decreased metabolism in association with blindness. Unidirectional metabolic decreases were observed in CB in most regions other than visual areas. (b) Similarly, the unthresholded *t*-maps with GMN indicated regionally bidirectional metabolic changes with blindness and unidirectional metabolic increases were observed in regions associated with vision. Based on validation of qCMR_glc_ to aCMR_glc_-HYD (Figures 1 and 2; Table 2), with and without GMN the global changes were essentially negligible. There were increases/decreases in visual/nonvisual areas, either with or without GMN. See Figure S6 for thresholded maps (Table 4).

Relative to the HAEC control group, the statistical *t*-maps for the disorders of consciousness groups (UWS, MCS, and EMCS), sedated groups (Des1%, Sev0.25%, and Sev0.5%), healthy participants with eyes open group (HAEO), and the group of congenitally blind subjects (CB), respectively, are shown in Figures 3 [Fig fig4] [Fig fig5]–6, respectively.

The UWS, MCS, and EMCS group members all had global declines, judging from the qCMR_glc_ *t*-maps (Figure 3(a)), consistent with the greatest decrease in subcortical regions. In contrast, judged from the GMN *t*-maps, the UWS, MCS, and EMCS group members had subcortical hypometabolism, whereas other regions had relative hypermetabolism (Figure 3(b)). Compared to the MCS and EMCS group members, the UWS group had areas of relative hypermetabolism in subcortical gray matter (Figure 3(b)).

The Sev0.25%, Sev0.5%, and Des1% groups all showed global metabolic decrease in the qCMR_glc_ *t*-maps (Figure 4(a)), with the Des1% group having less of a decline in white matter than the Sev0.25% and Sev0.5% groups. In contrast, using GMN *t*-maps, the Sev0.25%, Sev0.5%, and Des1% groups all had a regional pattern of both hypometabolism and hypermetabolism (Figure 4(b)). The Des1% group had relative hypermetabolism of deep brain regions, whereas the Sev0.25% and Sev0.5% groups had common patterns of bidirectional change that were most pronounced and of greatest spatial extent in the Sev0.5% group.

The HAEO group had diffuse global increases, estimated from the qCMR_glc_ *t*-maps with the greatest increase in the occipital cortex (Figure 5(a)), while GMN *t*-maps in contrast showed relative white matter hypermetabolism and gray matter hypometabolism, except in the visual cortex, which had hypermetabolism (Figure 5(b)).

Only the CB group members had brain regions of both metabolic increases and decreases (albeit of smaller magnitudes) in the qCMR_glc_ *t*-maps, with the increases mainly in the visual cortex and the decreases beyond the visual cortex (Figure 6(a)). This pattern was repeated when the GMN *t*-maps revealed large domains of hypometabolic and hypermetabolic cortices, with vision areas showing the strongest relative hypermetabolism (Figure 6(b)). While in the qCMR_glc_ *t*-maps the hypometabolic (green in Figure 6(a)) and hypermetabolic (red in Figure 6(a)) regions revealed homogenous activities, in the GMN *t*-maps the hypometabolic (blue and green in Figure 6(b)) and hypermetabolic (red and yellow in Figure 6(b)) regions showed heterogeneous activities.

The hot and cold colors in Figures 3 [Fig fig4] [Fig fig5]–6 enabled visualization of the effect upon thresholding. However, we could not apply the same statistical threshold across all conditions because of large variation of groups' sizes (Table 1). Thus, we used thresholding as a means to reveal positive and negative clusters with GMN versus qCMR_glc_ images, when compared to the control condition of eyes closed (Table 4). With disorders of consciousness (Figure S3), for the qCMR_glc_ images there were only large-sized negative clusters (>98% of voxels), whereas in GMN images there were many smaller-sized negative (6–7% of voxels) and positive (0.1–14% of voxels) clusters. With anesthesia sedation (Figure S4), for the qCMR_glc_ images there were only large-sized negative clusters (71–96% of voxels), whereas in GMN images there were many smaller-sized negative (0.2–13% of voxels) and positive (0.3–11% of voxels) clusters. With eyes open in awake/healthy (Figure S5), for the qCMR_glc_ images there was only one large-sized positive cluster (60% of voxels), whereas in GMN images there were many smaller-sized negative (2–20% of voxels) and positive (0.1–9% of voxels) clusters. With congenitally blind (Figure S6), for the qCMR_glc_ images there was only one small-sized positive cluster and two small-sized negative clusters (each 1% of voxels), whereas in GMN images there was one large-sized negative cluster (45% of voxels) and three smaller-sized positive clusters (0.2–27% of voxels) clusters. In brief, the thresholded *t*-maps showed that the number of positive/negative clusters in the GMN images were much greater (Table 4). Thus, all groups, except CB, had globally unidirectional metabolic offsets in qCMR_glc_ *t*-maps, whereas regionally bidirectional differences were seen for all groups in GMN *t*-maps. In addition, the hypometabolism and hypermetabolic regions identified by GMN *t*-maps depict metabolic changes that are substantially smaller in magnitude (i.e., 3–6 times) than the global differences captured by the qCMR_glc_ *t*-maps (Figure S7).

## 4. Discussion

Absolute quantification of brain glucose metabolism with FDG-PET requires continuous arterial blood sampling throughout the imaging procedure [1, 39]. As arterial blood sampling in clinical settings is difficult or logistically impossible, alternative approaches are commonly used to determine relative differences among groups or conditions. The complementary approaches for quantitative PET generally involve a form of interindividual normalization, based on the ratio of dose injected and body weight as a proportional index of arterial input [40] or on the average uptake in whole brain, gray matter, or a preselected reference region inside [10] or outside [33] the brain. Moreover, there are considerations of arterialized venous sampling [41, 42] and image-derived input functions [43, 44]. The validity of any normalization approach relies on specific assumptions that usually are not readily testable, such as the linearity of the relationship between body weight and distribution volume, the expected range of metabolic changes (i.e., regional versus global), or the validity of a chosen reference region for the population being examined.

Here, we used a new validated method for deriving quantitative baseline metabolic activity from FDG-PET without individual normalization [37], but where the quantified measure of CMR_glc_ (qCMR_glc_) for the HAEC condition was compared to the absolute CMR_glc_ (aCMR_glc_) from Hyder et al. (aCMR_glc_-HYD), also for the HAEC condition [36]. The process consisted of two steps. First, an intensity transformation was computed on a per-site basis for all HAEC datasets, using the Jensen-Shannon divergence method [38], to match the distribution of voxel intensities to the aCMR_glc_-HYD database [36]. This enabled the original tissue-radioactivity values for each HAEC group to be converted to aCMR_glc_ units on a per-site basis. This procedure also created a per-site intensity transformation that maps the original PET radioactivity counts to qCMR_glc,_ which can be used to convert radioactivity values for other conditions (i.e., conditions without lesions) scanned at that site using the same scanning parameters into aCMR_glc_ units. Finally, we validated this procedure by comparing qCMR_glc_ to aCMR_glc_, on a voxel-by-voxel basis using Pearson correlation and Euclidean distance for all gray matter regions between the two datasets.

Our goal was to compare glucose metabolism measured by PET from a large number of conditions, including specific levels of sedation depth induced by anesthesia, several levels of disorder of consciousness, awake/healthy with eyes open, and congenital blindness. Each cohort included a control group of healthy, awake individuals resting with eyes closed, which were all comparable across sites. The validated qCMR_glc_ group data led to new insights into the effects of GMN on the detection and interpretation of global versus regional metabolic estimates.

The qCMR_glc_ maps for all states (except the congenitally blind) revealed significant global differences relative to the eyes closed control group, which ranged in magnitude from ~10% increase for the awake, eyes open group to ~60% decrease in the unresponsive wakefulness syndrome. These global changes of qCMR_glc_ are in good agreement with previous findings of changes with eyes open versus eyes closed states [45, 46], congenitally blind versus healthy sighted subjects [47, 48], effects of halogenated anesthetics [49–52], and findings in disorders of consciousness [35, 53]. Specifically, various anesthetics and disorders of consciousness have largely reported globally depressed metabolism compared to the healthy condition (see references within [19, 33]). After GMN, these large global changes were absent from the GMN *t*-maps due to regression to the mean value. Consequently, the GMN *t*-maps showed patterns of regional increase and decrease in metabolism among different states, suggesting that significant global information is not captured with the GMN procedure. Although increases/decreases were observed in congenitally blind with/without GMN, both the hypometabolic and hypermetabolic regions showed heterogeneous activities upon GMN. These results suggest that global normalization puts an overemphasis on regional differences.

### 4.1. GMN Eliminates Global Metabolic Changes across States

In all conditions other than congenitally blind, we found globally unidirectional changes of qCMR_glc_ estimates compared to the control group, with metabolic differences among states distributed within a large range (i.e., 0.14 to 0.34 *μ*mol/g/min). In sharp contrast, GMN yielded regional increases and decreases compared to the eyes closed control group, with relative metabolic rate differences among states distributed within a narrow range (i.e., ±0.05 *μ*mol/g/min). These results suggest that the global component of FDG-PET images contains state-dependent metabolic information that is lost upon GMN. Moreover, the present work shows that the hypometabolism and hypermetabolic regions revealed by GMN depict metabolic changes that are substantially smaller in magnitude than the inherent global metabolic differences. Although the regional pattern of deviations from the global mean of normalized FDG conveys important information about metabolic networks, exclusion of the global mean can yield different interpretations such as the regionally increased metabolic activity to disease states, a concern previously raised in the context of neurodegenerative diseases [12, 54].

However, when absolute differences are of small magnitude and regionalized, as in the present comparison of congenitally blind to the sighted control group, images with and without GMN showed very similar patterns of hypometabolism and hypermetabolic areas. In this particular case, the GMN procedure exposed the differences only after removal of interindividual global variations, without any penalty for misrepresentation of the magnitude of the differences. Overall, these comparisons, especially with that of the congenitally blind group versus the other groups, strongly suggest that there are new insights to be gained by inclusion of both absolute and GMN analysis for PET-FDG data of neuropsychiatric and neurodegenerative diseases.

### 4.2. Study Limitations and Future Directions

The main limitation of the current study is the acquisition of PET-FDG images from multiple sites that were calibrated to produce qCMR_glc_ comparisons across the different groups, thereby limiting the statistical significance of state-dependent variations. The high similarity between qCMR_glc_ in the resting awake eyes closed (control) state across five different sites, which were nearly identical to aCMR_glc_ region-to-region variations, suggests that qCMR_glc_ maps from the different sites indeed were comparable. While the qCMR_glc_ measure proved stable on a per-group basis, this report did not investigate its validity on a per-subject basis, specifically for conditions with brain lesions. Future FDG-PET studies quantified with arterial sample inputs can improve aCMR_glc_ estimates to reduce the intersubject variation among groups. Notwithstanding this conclusion, the present qCMR_glc_ estimates had excellent correlations among all five control groups in this study. In the brain regions least vulnerable to effects of partial volume effects (i.e., regions defined by wide swathes of cortex with net spillover to and from adjacent tissues due to the inherent spatial smoothness of PET data is low), gray matter qCMR_glc_ exceeded the estimates in white matter by 2–3-fold for the control state, in good agreement with prior studies [19, 55, 56]. However, different magnetic resonance spectroscopy (MRS) methods (e.g., ^17^O MRS, ^31^P MRS, ^13^C MRS) and calibrated fMRI can be used to obtain absolute maps of CMR_glc_ or CMR_O2_ across these tissues for further validation [57].

Another concern of this study is the small sample sizes in three out of four sites and which limited the types of image analysis techniques we could employ. Variations in the Pearson correlation and the Euclidean distance for comparing across the control groups could be due to the multicenter data being heterogeneous (e.g., data acquired with different PET cameras). This and other weaknesses of the study limit the validity of the qCMR_glc_ method for imaging others brain disorders (e.g., Alzheimer's disease and Parkinson's disease) at this stage, and thus, this procedure could be considered if aCMR_glc_ comparisons are made for groups imaging across different brain states and/or PET scanners.

As noted above, qCMR_glc_ for gray and white matter is sensitive to partial volume effects because the thickness of the cerebral cortex is close to the spatial resolution delivered by current PET instruments [58]. Higher spatial resolution, such as that from the Siemens HRRT used to acquire FDG-derived images in the USA and Denmark sites, as well as improved MRI-based detection of cortical thickness, will provide better partial volume correction [59], propagating to increased accuracy of global versus regional metabolic differences measured across disease states. Using an HRRT scanner with sufficient resolution to measure the PET signal from the human globus pallidus, Borghammer et al. [14] employed reference cluster normalization to report that only the globus pallidus showed significant hypermetabolism in Parkinson's disease. A review of 2-deoxy-glucose studies of rodent and nonhuman primate models of Parkinson's disease showed that the globus pallidus most consistently reported true hypermetabolism [60]. Subsequent studies have dealt with the issue of normalization as raised by Borghammer et al. [14]. Dhawan et al. [61] reported that the spatial covariance pattern was not induced by reductions in global activity, opposing Borghammer et al. [14], whereas Dukart et al. [62] examined usefulness of different normalization procedures, and their findings do not contradict Borghammer et al. [14]. Since the sample sizes in some of our groups were small, we could not compare the results from different types of normalization methods (e.g., reference cluster and data-driven) and instead chose to compare effects on PET data with and without GMN.

Based on our results, and previous work, we thus caution that comparing any disease with the healthy condition should not begin with the assumption that global changes do not exist or, if they do exist, that they are not relevant to alterations in brain function (and behavior). We observed global changes across the entire brain without GMN in nearly all the states examined herein, but with GMN, these global changes were diminished showing bidirectional changes in all conditions examined. In one condition, the global changes appeared quite similar with and without GMN, but there was no assumption made about global or regional changes despite both analysis methods revealing nearly the same regional changes. Existing statistical analysis methods can undermine the relevance of global changes. For example, the Gaussian random field theory is optimized to detect small spatial regions of activity differences, defining “signal” as a limited spatial region whereas “noise” is defined as the background in large swathes of tissue. Also, if the global mean is used as a nuisance variable in techniques such as analysis of covariance, global changes will, by definition, be obscured. While our current work is focused on univariate analysis, if one includes the global signal as a “covariate of no interest,” it does not matter whether the analysis is univariate or multivariate, the procedure still effectively removes global effects. For example, if the total signal (and underlying neuronal activity) from all brain regions were reduced by a factor of 2, both univariate and multivariate analysis would conclude no difference across regions, unless an absolute measure of the global component was included. The basic idea that the global signal should not be discarded applies to both univariate and multivariate analysis. Thus, to adequately measure signal versus noise in global as well as regional brain metabolism with the highest level of confidence, higher-sensitivity imaging methods are needed in combination with different statistical analysis methods, which are beyond the scope of the current work and are issues for future studies.

## 5. Conclusions

At present, analysis of PET data generally ignores the global baseline signal. However, both the baseline neuronal activity and the requisite energy demands supporting the activity of the cerebral cortex of awake humans are substantial [25, 26, 63]. Removing the global PET signal prior to comparison with the resting awake eyes closed (control) state exposed regionally bidirectional metabolic effects, along with some regional changes observed upon normalization. Improper use of global signal normalization may thus lead to the incorrect assignment of elevated metabolism to regions and, by inference, the presence of elevated neuronal activity despite an impaired state of consciousness. Conversely, the approach used here (i.e., without GMN) not only preserved the global alteration caused by sedation and consciousness disorders but also detected localized abnormalities in the context of the congenitally blind. In light of the current findings, we recommend that the baseline metabolic activity be included in the analysis of PET neuroimaging data, and only then is it possible to discern global and regional metabolic differences between healthy and diseased states.

## Figures and Tables

**Table 1:** Details of different groups imaged at the various sites (Germany, USA, Denmark, and Belgium; see text for details).

PET imaging site	Experimental group	Control group
Site number 1, Germany (Technical University of Munich)	HAEO (*n* = 11)	HAEC_GER_ (*n* = 11)

Site number 2, USA (University of California, Irvine)	^∗^ Sev0.25% (*n* = 8)	^∗^ HAEC_sev_ (*n* = 8)
	^∗^ Sev0.5% (*n* = 8)	
	^∗^ Des1% (*n* = 7)	^∗^ HAEC_des_ (*n* = 7)

Site number 3, Denmark (Rigshospitalet, Copenhagen University Hospital)	CB (*n* = 7)	HAEC_DEN_ (*n* = 7)

Site number 4, Belgium (University Hospital of Liege)	UWS (*n* = 65)	HAEC_BEL_ (*n* = 28)
	MCS (*n* = 65)	
	EMCS (*n* = 17)	

∗ indicates that both absolute CMR_glc_ (aCMR_glc_) and quantified CMR_glc_ (qCMR_glc_) were obtained from the USA site, enabling comparison between them (see Figures 1(b) and 1(c)). aCMR_glc_: absolute CMR_glc_ with blood sampling of the tracer FDG supply to the brain; qCMR_glc_: calibration of quantified comparing qCMR_glc_ with aCMR_glc_ for HAEC only eqs. (1 and 2); HAEC: healthy people awake with eyes closed (control condition); HAEO: healthy people awake with eyes open; Des1%: healthy people sedated with 1% desflurane; Sev0.25%: healthy people sedated with 0.25% sevoflurane; Sev0.50%: healthy people sedated with 0.5% sevoflurane; CB: awake people with congenital blindness; UWS: patients who were unresponsive wakefulness syndrome; MCS: patients who were in a minimally conscious state; EMCS: patients who emerged from MCS.

**Table 2:** Results of quantified CMR_glc_ (qCMR_glc_) from Figure 1(a), where HAEC groups from different sites are compared to absolute CMR_glc_ from Hyder et al. [36] (aCMR_glc_-HYD). The upper triangular half is Pearson correlation (italicized), whereas the lower triangular half is Euclidean distance (non-italicized). In the table *p* = 0 for all entries. The Pearson correlations were highly significant, and all the Euclidean distances were less than even one despite a total of 41 dimensions' means. The high similarity between qCMR_glc_ in the HAEC groups measured at different sites indicates that comparisons between sites are possible using our procedure for quantified CMR_glc_.

					qCMR_glc_		
		aCMR_glc_-HYD	HAEC_des_	HAEC_sev_	HAEC_GER_	HAEC_DEN_	HAEC_BEL_
	aCMR_glc_-HYD		*0.82*	*0.771*	*0.908*	*0.878*	*0.912*
	HAEC_des_	0.233		*0.993*	*0.763*	*0.973*	*0.942*
	HAEC_sev_	0.246	0.0401		*0.712*	*0.956*	*0.905*
qCMR_glc_	HAEC_GER_	0.213	0.202	0.228		*0.851*	*0.876*
	HAEC_DEN_	0.243	0.0851	0.11	0.154		*0.946*
	HAEC_BEL_	0.177	0.108	0.137	0.135	0.111	

**Table 3:** Relationship of quantified CMR_glc_ (qCMR_glc_) in gray and white matter of the human brain, comparing different states to the control condition, as assessed by linear regression analysis with (intercept = 0) and without (intercept ≠ 0) an intercept at the origin. See Table 1 for abbreviations of conditions. See Figures S1 and S2 for details on intercept = 0.

	Intercept ≠ 0			Intercept = 0	
HAEC versus	Slope	Intercept	*R* ^2^	Slope	*R* ^2^
*Gray matter*					
HAEO	1.12	0.00	0.93	1.12	0.93
CB	0.87	0.03	0.87	0.97	0.89
Sev0.25%	0.87	0.02	0.98	0.92	0.98
Sev0.5%	0.79	0.02	0.98	0.85	0.97
Des1%	0.73	0.04	0.95	0.86	0.94
EMCS	0.54	0.03	0.8	0.64	0.83
MCS	0.49	0.04	0.73	0.6	0.78
UWS	0.26	0.07	0.65	0.46	0.73
*White matter*					
HAEO	1.10	0.00	0.95	1.08	0.95
CB	0.90	0.02	0.92	0.98	0.93
Sev0.25%	0.88	0.01	0.99	0.99	0.92
Sev0.5%	0.81	0.01	0.98	0.88	0.98
Des1%	0.77	0.03	0.97	0.91	0.96
EMCS	0.61	0.02	0.81	0.71	0.84
MCS	0.59	0.02	0.73	0.69	0.77
UWS	0.35	0.05	0.64	0.57	0.73

**Table 4:** Thesholding *t*-maps in Figures 3 [Fig fig4] [Fig fig5]–6 revealed unidirectional and bidirectional changes, which is illustrated in terms of the number of positive (*P*) and negative (*N*) clusters, where they, respectively, correspond to areas of higher and lower intensities compared to control. See Table 1 for abbreviations of conditions. The positive clusters in the thresholded GMN (P_GMN_) versus qCMR_glc_ (P_qCMR_) *t*-maps were 8 times greater, whereas negative clusters in the thresholded GMN (N_GMN_) versus qCMR_glc_ (N_qCMR_) *t*-maps were 2 times greater. Similarity between thresholded GMN and qCMR_glc_ *t*-maps was assessed by several metrics: (i) the total number of clusters given by the sum of P and N clusters, for qCMR_glc_ (T_qCMR_ = P_qCMR_ + N_qCMR_) and GMN (T_GMN_ = P_GMN_ + N_GMN_) thresholded *t*-maps; (ii) the difference between the P clusters (D_P_) for GMN and qCMR_glc_ thresholded *t*-maps (D_P_ = P_GMN_ − P_qCMR_); (iii) the difference between the N clusters (D_N_) for GMN and qCMR_glc_ thresholded *t*-maps (D_N_ = N_GMN_ − N_qCMR_). Analysis shows that T_GMN_ was about 4 times greater than T_qCMR_, whereas both D_P_ and D*_N_* were greater than 0, signifying that GMN thresholded *t*-maps consistently revealed more bidirectional changes. For thresholded *t*-maps, see Figure S3 for EMCS, MCS, and UWS versus control (HAEC); Figure S4 for Sev0.25, Sev0.5, and Des1 versus control (HAEC); Figure S5 for HAEO versus control (HAEC); and Figure S6 for CB versus control (HAEC).

Condition versus control	Threshold (*t*-value)	Number of clusters				Similarity between GMN and qCMR_glc_			
		P_qCMR_	N_qCMR_	P_GMN_	N_GMN_	T_qCMR_	T_GMN_	D_P_	D_N_
EMCS	4	0	1	2	1	1	3	2	0
MCS	4	0	1	5	1	1	6	5	0
UWS	4	0	1	2	1	1	3	2	0
Sev0.25%	2	0	1	1	2	1	3	1	1
Sev0.5%	2	0	1	0	4	1	4	0	3
Des1%	2	0	1	2	3	1	5	2	2
HAEO	1	1	0	3	2	1	5	2	2
CB	0.5	1	2	3	1	3	4	2	−1
Mean ± standard deviation		0.3 ± 0.5	1.0 ± 0.5	2.3 ± 1.5	1.9 ± 1.1	1.3 ± 0.7	4.1 ± 1.1	2.0 ± 1.4	0.9 ± 1.4

## Abbreviations

BOLD: Blood oxygen level-dependent
CBF: Cerebral blood flow
CMR_glc_: Cerebral metabolic rate of glucose metabolism
CMR_O2_: Cerebral metabolic rate of oxygen metabolism
GMN: Global mean normalization
MNI: Montreal Neurological Institute
PET: Positron emission tomography.

## Supplementary Materials

Supplementary Materials Figure S1: voxel-to-voxel correlations of qCMRglc in gray matter of the human brain. Figure S2: voxel-to-voxel correlations of qCMRglc in white matter of the human brain. Figure S3: thresholded *t*-maps of metabolic variations in disorders of consciousness. Figure S4: thresholded *t*-maps of metabolic variations in anesthetic sedation. Figure S5: thresholded *t*-maps of metabolic variations with eyes open. Figure S6: thresholded *t*-maps of metabolic variations in congenital blindness. Figure S7: magnitude of metabolic variations with and without GMN. Table S1: description of human gray matter regions.
