# Supplementary material for: Impact of Global Mean Normalization on Regional Glucose Metabolism in the Human Brain
Source: Neural Plast. 2018 Jun 12;2018:6120925. doi: 10.1155/2018/6120925 (PMC6020504; doi:10.1155/2018/6120925)
Supplement: Supplementary Materials — Figure S1: voxel-to-voxel correlations of qCMRglc in gray matter of the human brain. Figure S2: voxel-to-voxel correlations of qCMRglc in white matter of the human brain. Figure S3: thresholded t-maps of metabolic variations in disorders of consciousness. Figure S4: thresholded t-maps of metabolic variations in anesthetic sedation. Figure S5: thresholded t-maps of metabolic variations with eyes open. Figure S6: thresholded t-maps of metabolic variations in congenital blindness. Figure S7: magnitude of metabolic variations with and without GMN. Table S1: description of human gray matter regions. [file 6120925.f1.pdf]

SUPPLEMENTARY MATERIALS

for

Impact of global mean normalization on regional glucose metabolism in the human brain

Kristian N. Mortensen,[1,2] Albert Gjedde,[2] Garth J. Thompson[1], Peter Herman[1], Maxime J. Parent[1], Douglas L. Rothman[1,3], Ron Kupers[2], Maurice Ptito,[2,4,5] Johan Stender[2,6], Steven Laureys[6], Valentin Riedl[7], Michael T. Alkire[8], Fahmeed Hyder[1,3]

[1] Department of Radiology & Biomedical Imaging and Magnetic Resonance Research Center, Yale University, New Haven, CT, USA

[2] Department of Neuroscience and Pharmacology, University of Copenhagen, Copenhagen, Denmark

[3] Department of Biomedical Engineering, Yale University, New Haven, CT, USA

[4] Chaire de Recherche Harland Sanders, School of Optometry, University of Montreal, Montreal, Canada

[5] Neuropsychiatry laboratory, Psychiatric Centre, Rigshospitalet, Copenhagen, Denmark

[6] GIGA-Consciousness, Coma Science Group, Université de Liège, Liège, Belgium

[7] Departments of Neuroradiology, Nuclear Medicine and Neuroimaging Center, Technische Universität München, München, Germany

[8] Department of Anesthesiology, University of California, Irvine, CA, USA

Figure S1. Voxel-to-voxel correlations of $qCMR_{glc}$ in gray matter of human brain.

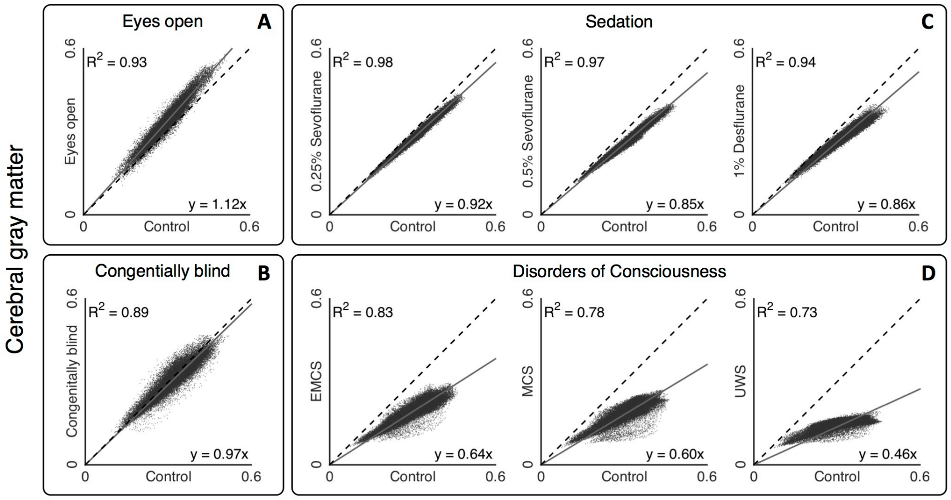

The horizontal axis represents the $qCMR_{glc}$ for the control group (i.e., normal awake sighted people with eyes closed; **Figure 2A, middle**), whereas the vertical axis displays the $qCMR_{glc}$ for the 8 other groups, which included **(A)** normal awake sighted people with eyes open **(Figure 2A, top)**, **(B)** awake people with congenital blindness **(Figure 2A, bottom)**, **(C)** normal people under sedation (i.e., 1% desflurane, 0.25% sevoflurane, 0.5% sevoflurane; **Figure 2B**), and **(D)** patients with disorders of consciousness (i.e., unresponsive wakefulness syndrome (UWS), minimally conscious state (MCS), emergence from MCS (EMCS); **Figure 2C**). All correlation coefficients, derived from all voxels in gray matter, were highly significant (i.e., R^2 ranging from 0.73 to 0.98), where lines for regression and identity are, respectively, shown with solid and dashed lines. **(A)** The normal eyes open group (vs. the control group) showed ~12% higher $qCMR_{glc}$. **(B)** The congenitally blind group (vs. the control group) showed 2-3% lower $qCMR_{glc}$. **(C)** The sedated groups (vs. the control group) showed ~8% lower $qCMR_{glc}$ with 0.25% sevoflurane, ~15% lower $qCMR_{glc}$ with 0.5% sevoflurane, and ~14% lower $qCMR_{glc}$ with 1% desflurane. **(D)** The disorders of consciousness groups (vs. the control group) showed ~36% lower $qCMR_{glc}$ with EMCS, ~40% lower $qCMR_{glc}$ with MCS, and ~54% lower $qCMR_{glc}$ with UWS. See **Table 3** for comparison of slopes and intercepts when the linear regression was conducted without forcing the intercept through the origin.

Figure S2. Voxel-to-voxel correlations of $qCMR_{glc}$ in white matter of human brain.

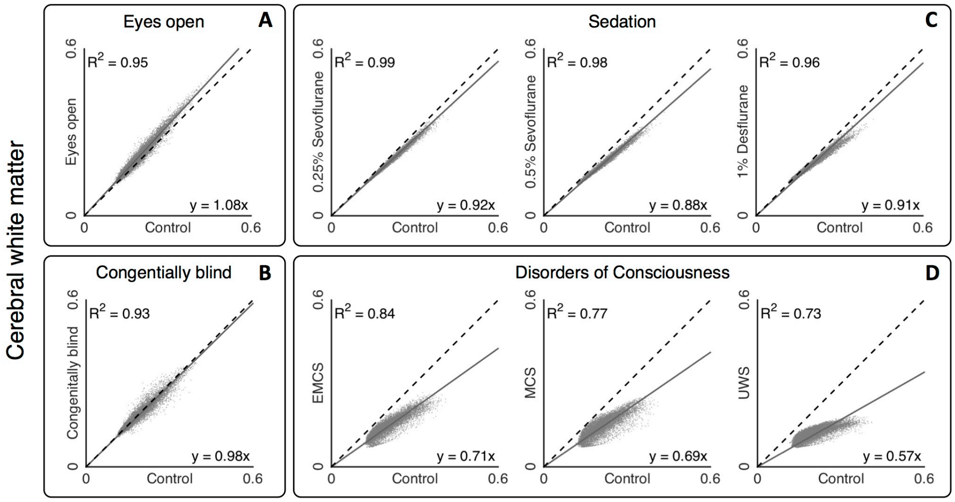

The horizontal axis represents the $qCMR_{glc}$ for the control group (i.e., normal awake sighted people with eyes closed; **Figure 2A, middle**), whereas the vertical axis displays the $qCMR_{glc}$ for the 8 other groups, which included **(A)** normal awake sighted people with eyes open (**Figure 2A, top**), **(B)** awake people with congenital blindness (**Figure 2A, bottom**), **(C)** normal people under sedation (i.e., 1% desflurane, 0.25% sevoflurane, 0.5% sevoflurane; **Figure 2B**), and **(D)** patients with disorders of consciousness (i.e., unresponsive wakefulness syndrome (UWS), minimally conscious state (MCS), emergence from MCS (EMCS); **Figure 2C**). All correlation coefficients, derived from all voxels in white matter, were highly significant (i.e., R^2 ranging from 0.73 to 0.99), where lines for regression and identity are, respectively, shown with solid and dashed lines. **(A)** The normal eyes open group (vs. the control group) showed ~8% higher $qCMR_{glc}$. **(B)** The congenitally blind group (vs. the control group) showed 1-2% lower $qCMR_{glc}$. **(C)** The sedated groups (vs. the control group) showed ~8% lower $qCMR_{glc}$ with 0.25% sevoflurane, ~12% lower $qCMR_{glc}$ with 0.5% sevoflurane, and ~9% lower $qCMR_{glc}$ with 1% desflurane. **(D)** The disorders of consciousness groups (vs. the control group) showed ~29% lower $qCMR_{glc}$ with EMCS, ~31% lower $qCMR_{glc}$ with MCS, and ~43% lower $qCMR_{glc}$ with UWS. See **Table 3** for comparison of slopes and intercepts when the linear regression was conducted without forcing the intercept through the origin.

Figure S3. Thresholded *t*-maps of metabolic variations in disorders of consciousness.

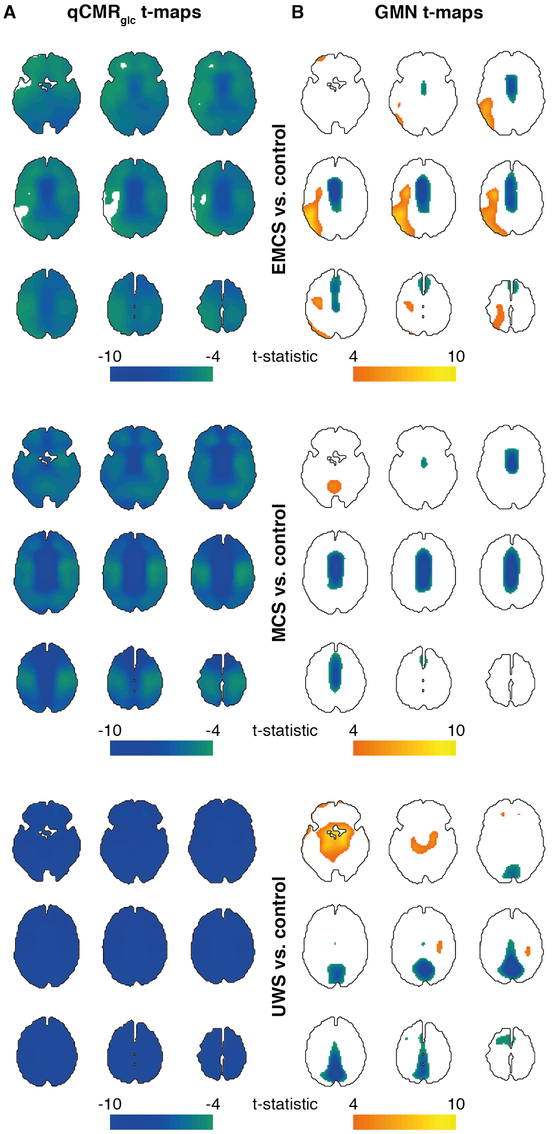

Spatial distributions of metabolic variations in patients with disorders of consciousness (i.e., UWS, MCS, EMCS in **Figure 2C** in the main text) vs. the control group (i.e., HAEC in **Figure 2A** in the main text), shown with respect to *thresholded* student's *t*-maps using **(A)** $qCMR_{glc}$ images and **(B)** GMN images. In $qCMR_{glc}$ images there are only large sized negative clusters (>98% of voxels), whereas in GMN images there are many smaller sized negative (6-7% of voxels) and positive (0.1-14% of voxels) clusters. The *unthresholded* student's *t*-maps are shown in **Figure 3** in the main text.

Figure S4. Thresholded *t*-maps of metabolic variations in anesthetic sedation.

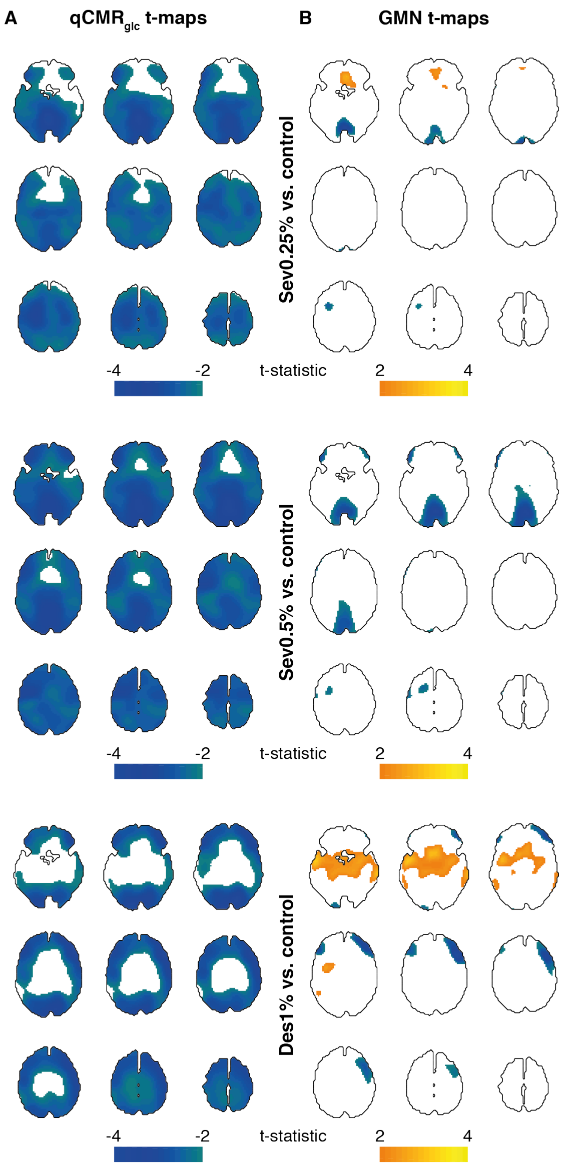

Spatial distributions of metabolic variations in patients with sedation (i.e., Des1%, Sev0.25%, Sev0.5% in **Figure 2B** in the main text) vs. the control group (i.e., HAEC in **Figure 2A** in the main text), shown with respect to *thresholded* student's *t*-maps using **(A)** $qCMR_{glc}$ images and **(B)** GMN images. In $qCMR_{glc}$ images there are only large sized negative clusters (71-96% of voxels), whereas in GMN images there are many smaller sized negative (0.2-13% of voxels) and positive (0.3-11% of voxels) clusters. The *unthresholded* student's *t*-maps are shown in **Figure 4** in the main text.

Figure S5. Thresholded *t*-maps of metabolic variations with eyes open.

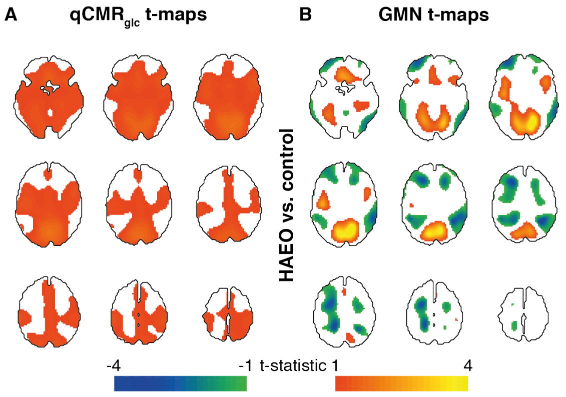

Spatial distributions of metabolic variations with healthy participants with eyes open (i.e., HAEO in **Figure 2B** in the main text) vs. the eyes-closed control group (i.e., HAEC in **Figure 2A** in the main text), shown with respect to *thresholded* student's *t*-maps using **(A)** $qCMR_{glc}$ images and **(B)** GMN images. In $qCMR_{glc}$ images there is only one large sized positive cluster (60% of voxels), whereas in GMN images there are many smaller sized negative (2-20% of voxels) and positive (0.1-9% of voxels) clusters. The *unthresholded* student's t-maps are shown in **Figure 5** in the main text.

Figure S6. Thresholded *t*-maps of metabolic variations in congenital blindness.

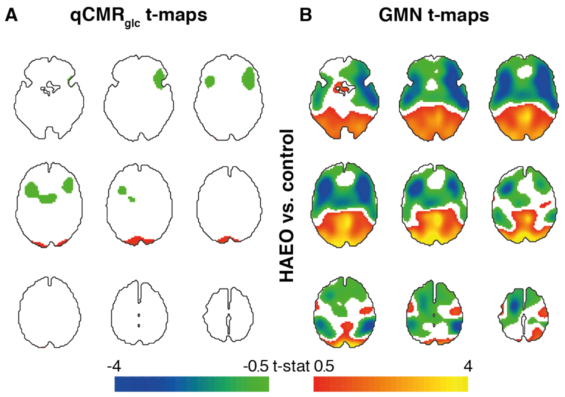

Spatial distributions of metabolic variations in the congenitally blind (i.e., CB in **Figure 2B** in the main text) vs. the eyes-closed control group (i.e., HAEC in **Figure 2A** in the main text), shown with respect to *thresholded* student's *t*-maps using **(A)** $qCMR_{glc}$ images and **(B)** GMN images. In $qCMR_{glc}$ images there is only one small sized positive cluster and two small sized negative clusters (each 1% of voxels), whereas in GMN images there is one large sized negative cluster (45% of voxels) and three smaller sized positive clusters (0.2-27% of voxels) clusters. The *unthresholded* student's t-maps are shown in **Figure 6** in the main text.

Figure S7. Magnitude of metabolic variations with and without GMN.

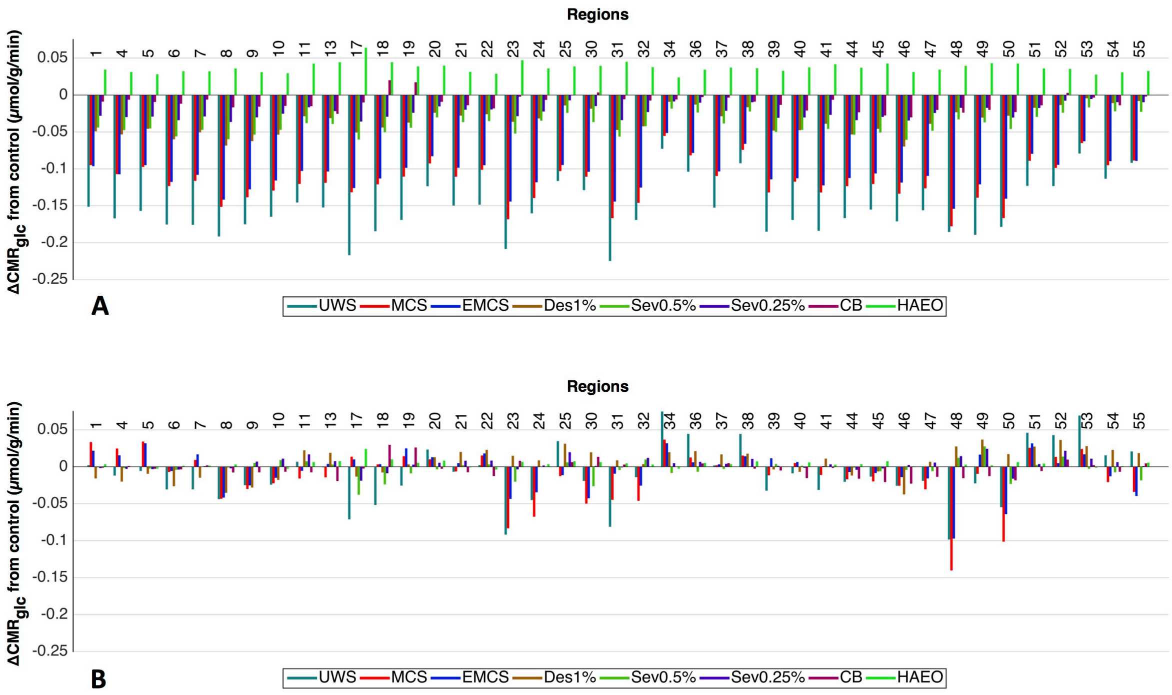

Magnitude of changes in glucose metabolism (ΔCMR_{glc}) across gray matter for different resting states in relation to the control group of healthy awake sighted people with eyes closed (i.e., HAEC in **Figure 1A**, middle) using **(A)** $qCMR_{glc}$ images and **(B)** GMN images. The horizontal axis represents Brodmann-like regions **(Table S1)**, whereas the vertical axis displays ΔCMR_{glc} for 8 groups (vs. the control group), which included healthy awake sighted people with eyes open (i.e., HAEO in **Figure 1A**, top), awake people with congenital blindness (i.e., CB in **Figure 1A**, bottom), normal people under sedation (i.e., Des1%, Sev0.25%, Sev0.5% in **Figure 1B**), and patients with disorders of consciousness (i.e., UWS, MCS, EMCS in **Figure 1C**). **(A)** With $qCMR_{glc}$ images for all groups, except CB, globally unidirectional ΔCMR_{glc} were detected, where the dynamic range of ΔCMR_{glc} from UWS to HAEO was ~0.3 μmol/g/min. **(B)** With GMN images for all groups regionally bidirectional ΔCMR_{glc} were detected, where the dynamic range of ΔCMR_{glc} from UWS to HAEO was ~0.1 μmol/g/min. See **Figures 3-6** and **Figures S3-S6** for unthresholded and thresholded *t*-maps, respectively.

Table S1. Description of Brodmann-like regions in the human gray matter.

Region	Size	Description of Region in terms of neuroanatomical identity (similar to Brodmann areas)
1	3.46%	Primary Somatosensory Cortex
4	2.35%	Primary Motor Cortex
5	0.65%	Somatosenory Association Cortex
6	8.68%	Premotor Cortex, Supplementary Motor Cortex
7	6.06%	Somatosenory Association Cortex, Precuneus
8	4.06%	includes Frontal Eye Fields
9	3.55%	Dorsolateral Prefrontal Cortex
10	6.26%	Anterior Prefrontal Cortex (most rostral part of superior and middle frontal gyri)
11	2.65%	Orbitofrontal Area (orbital and rectus gyri, plus the rostral part of the superior frontal gyrus)
13	1.95%	Insular Cortex
17	1.57%	Primary Visual Cortex (V1)
18	6.28%	Secondary Visual Cortex (V2)
19	5.71%	Associative Visual Cortex (V3)
20	2.55%	Inferior Temporal Gyrus
21	3.43%	Middle Temporal Gyrus
22	2.63%	Superior Temporal Gyrus, of which the caudal part is considered to contain the Wernicke's area
23	1.69%	Ventral Posterior Cingulate Cortex
24	1.18%	Ventral Anterior Cingulate Cortex
25	0.22%	Subgenual Cortex
30	0.33%	Part of Cingulate Cortex
31	1.84%	Dorsal Posterior Cingulate Cortex
32	1.37%	Dorsal Anterior Cingulate Cortex
34	0.07%	Anterior Entorhinal Cortex (on the parahippocampal gyrus)
36	1.80%	Parahippocampal Cortex (on the parahippocampal gyrus)
37	4.91%	Fusiform Gyrus
38	3.05%	Temporopolar Area (most rostral part of the superior and middle temporal gyri)
39	4.97%	Angular Gyrus, considered by some to be part of Wernicke's area
40	3.50%	Supramarginal Gyrus, considered by some to be part of Wernicke's area
41	1.13%	Primary and Auditory Association Cortex
44	2.46%	Pars Opercularis, part of Broca's area
45	1.30%	Pars Triangularis Broca's area
46	1.00%	Dorsolateral Prefrontal Cortex
47	2.59%	Inferior Prefrontal Gyrus
48	0.96%	Caudate
49	0.99%	Putamen
50	1.28%	Thalamus
51	0.15%	Globus Pallidus
52	0.04%	Nucleus Accumbens
53	0.41%	Amygdala
54	0.83%	Hippocampus
55	0.10%	Hypothalamus

Regions are some well-defined gray matter regions based on neuroanatomy. Shown above are 41 regions, their description in terms of the underlying neuroanatomy, and size in relation to the entire gray matter space.
